# Nocturia and frailty in older adults: a scoping review

**DOI:** 10.1186/s12877-024-05049-3

**Published:** 2024-06-06

**Authors:** Yulia Komleva, Maik Gollasch, Maximilian König

**Affiliations:** 1 Klinik und Poliklinik für Innere Medizin D–Geriatrie, Universitätsmedizin Greifswald, Walther-Rathenau-Str. 49, 17475 Greifswald, Germany; 2 Altersmedizinisches Zentrum, Kreiskrankenhaus Wolgast, Wolgast, Germany

**Keywords:** Nocturia, Frailty, Older adults, Geriatrics, Sleep disturbance, Circadian

## Abstract

**Background:**

More than one in two older people wake up several times a night to urinate. Far from being a minor inconvenience, nocturia is associated with poor health outcomes. Given the importance of sleep as a foundation for resilience and healthy ageing, nocturia may promote the development of frailty, which is inextricably linked to physical decline, disability, and mortality.

The aim of this scoping review was to collate published evidence on the relationship between nocturia and frailty, using the methodological framework of Arksey and O'Malley, together with the Joanna Briggs Institute methodology as guidance (OSF registration: osf.io/d5ct7).

**Methods:**

Relevant publications were retrieved via PubMed, Embase, the Cochrane Library and Google Scholar. The Rayyan tool was used to facilitate the screening process. Data were extracted by two independent reviewers. 250 publications were initially identified, of which 87 met the eligibility criteria.

**Results:**

Most of the evidence came from cross-sectional studies, most of which had been published within the last 5 years. The researchers were diverse, with 27% having a geriatric background. Only few publications established a clear association between nocturia and frailty. Other topics included: the association between nocturia and poor sleep quality and duration; the association between sleep and frailty; the association between frailty, multimorbidity, and age-related changes in the lower urinary tract.

**Conclusion:**

The findings emphasize the increasing interest and interdisciplinary nature of research into the relationship between frailty, nocturia, lower urinary tract symptoms, and sleep disturbances. Further research is required to enhance understanding, establish causality, and identify potential therapeutic approaches.

**Supplementary Information:**

The online version contains supplementary material available at 10.1186/s12877-024-05049-3.

## Introduction

The growth in both the size and the proportion of older persons in the population represents a major challenge to modern societies, with a substantial burden of disabling physical and cognitive impairments resulting from age-related diseases and geriatric syndromes, such as frailty, a state of depleted reserve capacity and increased risk of adverse health outcomes [1–3]. The prevalence of frailty in older adults ranges from 8 to 16% [4]. Its etiology is still characterized by a marked lack of comprehensive understanding [5]. Currently, frailty is thought to result from a complex interplay of various risk factors, including demographic and social factors such as advanced age, and educational attainment, as well as clinical determinants such as multimorbidity, obesity, and sleep disturbances, lifestyle factors such as physical inactivity, and biological underpinnings such as inflammation and endocrine dysregulation [6]. As there are no specific treatments, the primary approach to managing frailty is to optimize the determinants that contribute to its occurrence and to treat underlying diseases (e.g. controlling diabetes, heart failure, addressing malnutrition, etc.) [7–10].

Interviewing pre-frail and frail geriatric patients about their biggest annoyances reveals that nocturia is a prevalent and burdensome problem. Indeed, up to 4 in 5 older adults wake up several times during the night to urinate. [11] Nocturia is defined by the International Continence Society as “the number of times an individual passes urine during their main sleep period, from the time they have fallen asleep up to the intention to rise from that period”. It is an under-recognized and under-reported issue. This is partly due to the fact, that the cause of nocturia often appears obscure, there is no straight-forward and standardized diagnostic approach, treatment options are limited, and its significance to clinicians is relatively low. This creates therapeutic nihilism and makes nocturia an unmet medical need [12].

However, nocturia is far from being a minor inconvenience. It inevitably impacts both the quantity and quality of sleep [13]. Sleep disturbances are any disruptions or abnormalities in normal sleep patterns (quality, quantity and timing). Sleep disorders, on the other hand, are specific medical conditions characterized by persistent and severe disturbances in sleep patterns that interfere with normal functioning. The most common type is insomnia, which is characterized by difficulty falling asleep, staying asleep, or both.

Given the significance of good sleep as a basis for resilience and healthy ageing, nocturia represents a significant problem that is not given enough recognition. As nocturia and frailty are both prevalent in the older population, this raises questions about the link between nocturia and frailty. Nocturia may be a risk factor for frailty, a consequence of frailty, a co-occurring health problem—with shared risk factors, or a factor that maintains frailty once it has occurred. [14] Although not a hallmark of the frailty phenotype by default, nocturia, like other geriatric syndromes may be regarded a consequence of cumulative declines in various physiological systems, such as hormonal, urological, renal, cardiovascular, and metabolic systems, just like in the case of the frailty phenotype. [15]

A cursory literature search showed that there are quite a few publications that deal with this topic. However, the literature base appeared fragmented and not yet comprehensively reviewed. The aim of this scoping review was therefore to compile and map the existing evidence on the relationship between nocturia and frailty in older adults and to gain a deeper understanding of the relationship, and from this to derive topics for future research projects to fill knowledge gaps or test therapeutic approaches.

## Methods and analysis

The scoping review followed the methodological framework introduced by Arksey and O’Malley in 2005 [16] and was conducted in accordance with the Joanna Briggs Institute (JBI) methodology [17]. We adhered to the Preferred Reporting Items for Systematic Reviews and Meta-analyses extension for scoping reviews (PRISMA-ScR) checklist [18]. The review protocol was registered in Open Science Framework (osf.io/d5ct7).

### Research questions

- What evidence is there?
- Where does the evidence come from?
- Does the available evidence allow for a clear model of the relationship?
- Are there treatments that target nocturia and have an effect on frailty?
- What are the current and future research topics in this area?

Using these research questions as guidance, we engaged in an iterative process that involved searching the literature, identifying search terms, developing, and refining our search strategy to identify relevant publications.

### Eligibility criteria

JBI’s Population, Concept, Context (PCC) framework was used to refine the scope of the review and develop the eligibility criteria. Due to the broad nature of this scoping review, the inclusion criteria were designed to capture a comprehensive range of research publications exploring the associations between nocturia, frailty, lower urinary tract symptoms (LUTS), and sleep in older women and men aged 65 years and above, across diverse settings and study designs. There were no restrictions as to the publication type, date or study design. Also, conference abstracts were included.

### Search strategy

An initial limited search of the Cochrane Library, PubMed and Embase, completed in August 2023, using the search terms "frailty" OR "frail" AND "older adults" OR "elderly individuals" OR "elderly people" AND "nocturia" OR "nocturnal polyuria" identified 55 unique articles, which were screened for inclusion in this scoping review. The keywords contained in the titles and abstracts of relevant articles, and the index terms were carefully selected to inform our full search strategy to capture all relevant literature pertaining to the association between nocturia and frailty. In this way, additional search terms were identified: "LUTS" OR "Lower urinary tract symptoms and "sleep disturbances".

A second search using all the identified keywords and index terms was undertaken across all included databases. In addition to the Cochrane Library, PubMed and Embase, we reviewed the first 250 results from Google Scholar for publications to be included. This has been shown to be a reasonable approach given the countless search results with this web search engine [19, 20].

467 records were identified. All identified citations were uploaded into the Rayyan tool and duplicates removed [21]. Then, titles and abstracts of the remaining 250 records were screened for inclusion in this scoping review.

**Fig. 1:**
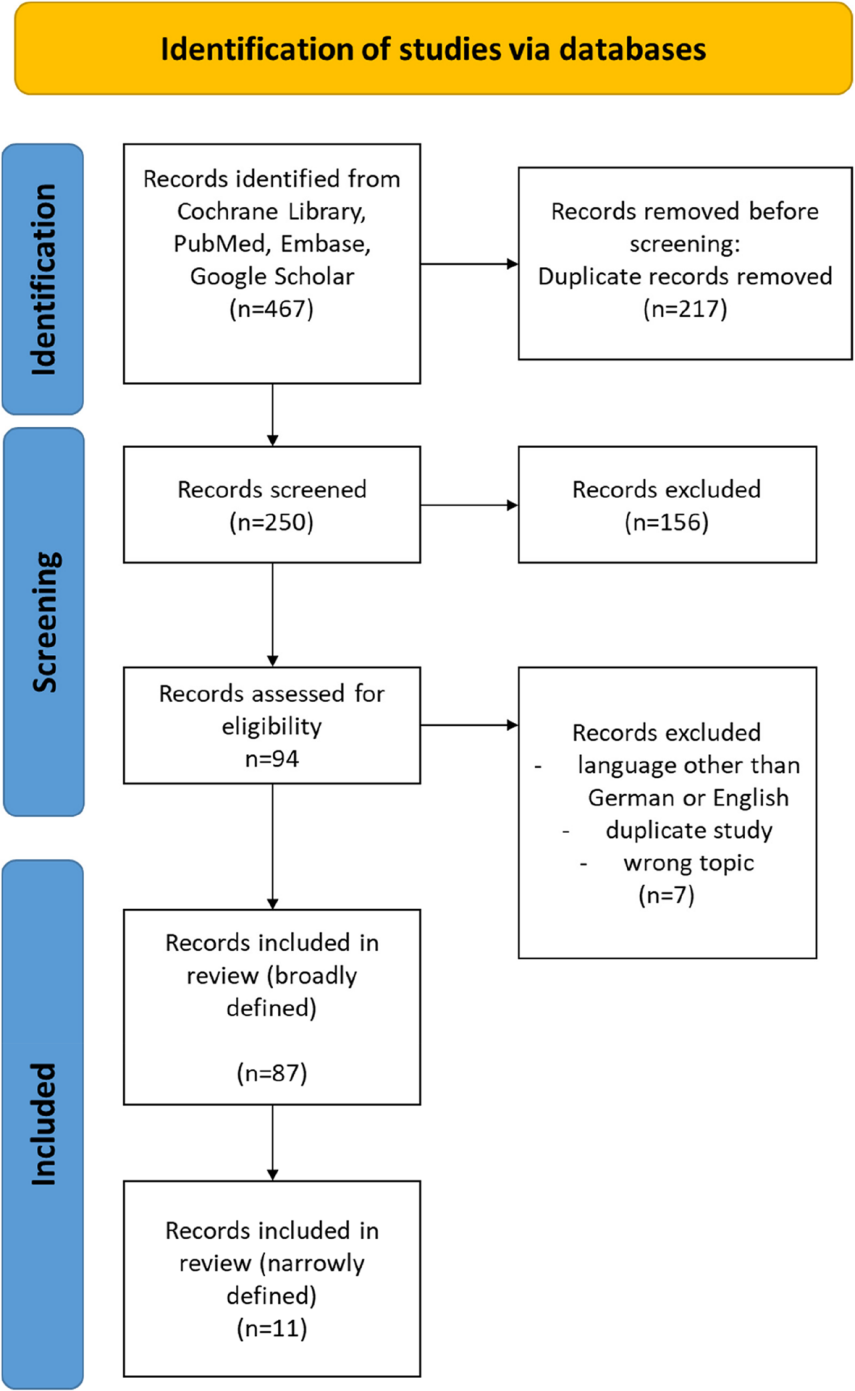
PRISMA-ScR flow diagram of the scoping review process

In the next step, 94 potentially relevant sources were retrieved in full and assessed in detail against the eligibility criteria. For conference abstracts, we conducted an additional targeted search to locate any existing full texts of published and accessible papers. Also, the reference lists of included studies were screened for additional studies of relevance. Studies published in English and German were eligible to be included. Studies up to and including September 2023 were considered. Two independent reviewers completed the screening. Any disagreements that arose between the reviewers at each stage of the selection process were resolved through discussion. The results of the search and the study inclusion process are presented in a PRISMA-ScR flow diagram [18] (Fig. 1).

### Data extraction

A data-extraction form, adapted from the JBI template, was jointly developed by two reviewers in an iterative process involving repeated pilot testing and modifications of the drafts to ensure the final form captures all relevant data (Supplementary Table 1). We extracted information on the type of study, the year of publication, the first authors’ disciplinary affiliations, sample characteristics, research design, whether frailty, nocturia, LUTS, and sleep were dealt with and the main outcome message [22].

### Quality appraisal

We critically appraised the studies’ strengths and limitations using the JBI checklist for quality appraisal.

### Collating, summarizing and reporting the results

**Fig. 2:**
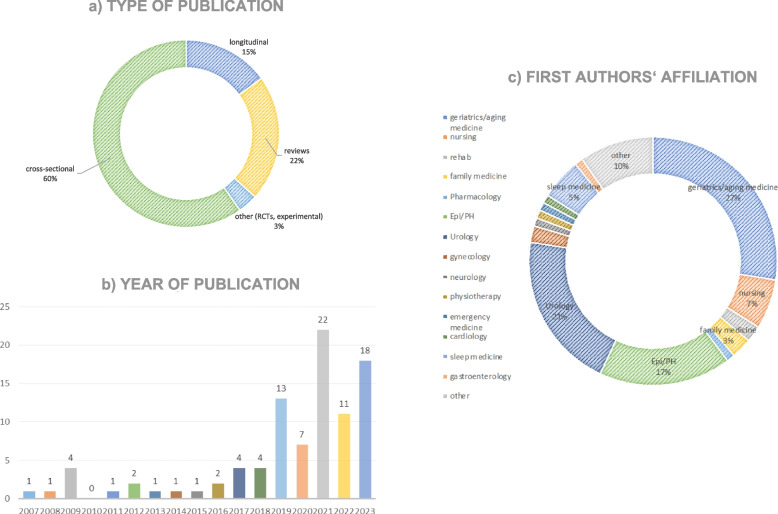
**a** The figure shows the distribution of publications by study type. **b** The figure illustrates the distribution of publications included in the review over time, indicating a significant increase in the number of publications on the topic in recent years. **c** The figure categorizes the reviewed publications based on the affiliation of their first authors

We collated and presented the results using a narrative summary, tables, and figures. Firstly, we looked at the literature in terms of study design, year of publication, and specialization of the first author (Fig. 2a-c). Then, we identified the main thematic complexes, to which we assigned each publication [23]. The evidence on each topic was summarized narratively. Furthermore, we attempted to embed nocturia and frailty in a unifying concept based on the reviewed publications, supplemented by selective, targeted literature research, and to derive possible intervention strategies from this.

### Ethics

This study involved neither human participants nor unpublished secondary data. Therefore, ethical approval was not required.

### Patient and public involvement

This work analyzed existing research studies, and therefore involved no patients or members of the public.

## Results

In the first step, the search revealed 55 hits. Screening of titles and abstracts suggested that it might be beneficial to expand the search to include the search strings "Sleep disturbances" AND "Frailty", and "LUTS" or “Lower urinary tract symptoms” AND "Frailty" to identify additional publications. As a result, further publications were identified (total *n* = 250). Titles and abstracts were screened by two independent reviewers for inclusion in this scoping review. 94 publications were retrieved in full text for examination, and 87 publications were eligible for inclusion. A flowchart of the selection process is presented in Fig. 1**.** All studies that were selected were collated in Supplementary Table 1. In this table we also indicated, which of the components of interest – nocturia, LUTS, sleep, and frailty—the respective publication dealt with.

According to the first authors’ disciplinary affiliations, many publications were led by a first author from a geriatric department, followed by the fields of urology and authors from cross-sectional disciplines, such as public health or epidemiology (Fig. 2a). Most publications were published within the last 5 years (Fig. 2b).

Most publications reported results from cross-sectional studies (*n* = 52). 22 percent were reviews or systematic reviews, and only 13 publications were based on longitudinal data (Fig. 2c).

**Fig. 3:**
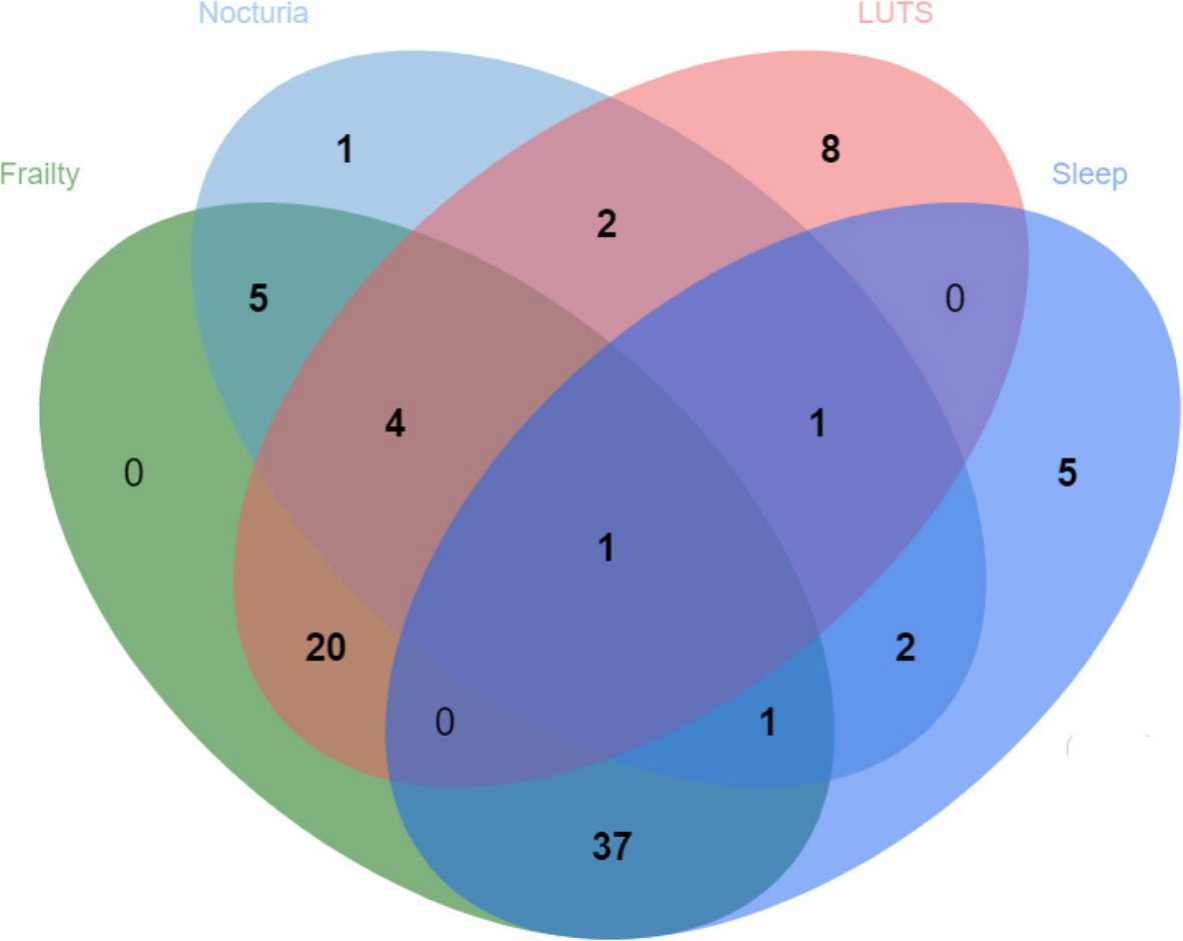
This Venn diagram uses overlapping ellipses to map the reviewed publications according to the respective thematic areas covered: frailty, sleep, nocturia, and lower urinary tract symptoms (LUTS)

A detailed content analysis revealed that the majority of the publications included in this scoping review focused on frailty (*n* = 68), 17 publications dealt with nocturia. Only 11 publications dealt with both, nocturia and frailty (Fig. 3 and Fig. 1).

The reviewed literature consistently showed that as people age, they increasingly experience frailty, nocturia, LUTS (including urinary incontinence), and sleep disturbances. Moreover, all entities appear to be closely interrelated in all directions. Five key themes emerged:

1. The association between nocturia and sleep.
2. The association between nocturia and frailty.
3. The association between sleep and frailty.
4. The association between frailty, comorbidity and urologic changes.
5. The importance of considering frailty in the management of patients with benign prostate hyperplasia (BPH) and LUTS.

### Nocturia and sleep

Five studies investigated the relationship between nocturia and sleep. According to several studies, nocturia is associated with an up to a sixfold increased risk of poor sleep quality [13]. For example, in a cross-sectional study of 723 adults in Japan, participants with two or more episodes of nocturia had significantly higher Pittsburgh-Sleep-Quality Index (PSQI) scores than those with fewer nocturia episodes [24]. Furthermore, an association was established between two or more episodes of voiding per night and higher Insomnia-Severity Index scores (OR = 2.3, 95% CI: 1.6–3.4) [25].

### Nocturia and frailty

Eleven publications were identified, that addressed the relationship between nocturia and frailty. While the majority reported some evidence of an association of nocturia with frailty, there were also negative results. E.g. Soma et al. showed in a cross-sectional study involving 710 individuals that nocturia (≥ 2 episodes per night) was associated with frailty, which was measured in different ways (Fried Frailty Phenotype (OR 2.15, 95% CI 1.42–3.24, *p* < 0.001), modified Frailty Index (OR 2.00, 95% CI 1.30–3.08, *p* = 0.002), and Frailty Discriminant Score (OR 2.09, 95% CI 1.45–3.02, *p* < 0.001)) [26]. The studies that could not verify an association between nocturia and frailty both had rather few participants: a cross-sectional multicenter study of 80 participants (mean age 88 ± 7 years, 77% women) [27], as well as another cross-sectional study with 158 men aged 65 years and above. The latter could not show any difference in nocturia prevalence between participants with a low, intermediate, and high frailty index [28]. In summary, the evidence was sparse and heterogeneous, especially with regard to the measurement of frailty, and in particular there were no longitudinal studies that allowed conclusions to be drawn about the temporal sequence and thus an indication of possible causality.

### Sleep and frailty

Significantly more evidence was available on the association between sleep and frailty (39 publications). Numerous studies have sought to dissect and understand the intricate connection between these two conditions. For example, research conducted by Yoshikoshi et al. revealed a significant association between increasing Athens Insomnia Scale scores and higher odds of physical frailty (OR 1.12)[29]. Similarly, a study by Fan et al. indicated a strong link between insomnia and the likelihood of frailty (OR 6.86) [30]. In particular, also longitudinal findings revealed that sleep disturbances were predictive of both the onset and progression of frailty during follow-up [31]. Several studies suggested that there is a difference between sleep duration and sleep quality in the association with frailty [6, 32, 33]. Arias-Fernández et al., but also other groups showed that poor self-reported sleep quality, but not sleep duration was associated with frailty [34]. In this sense, poor sleep quality, defined by a PSQI above 5, was associated with increased odds for both frailty (OR = 1.78, 95% CI 1.19–2.66) and pre-frailty (OR = 1.51, 95% CI 1.20–1.90) [35]. Another study showed that a PSQI above 7 was associated with 2.68-fold increased odds of frailty (OR 2.68, 95% CI 1.04-6.91) [36].

Also insomnia, i.e. difficulty initiating sleep or maintaining sleep, was shown to be associated with frailty. [37] Frail older adults were considerably more prone to experiencing insomnia compared to non-frail individuals, with rates of 36.4% versus 8.8% [38]. However, likewise there seems to be a difference between sleep-onset and sleep-maintenance insomnia, the latter showing less consistent associations with frailty [39]. Tang et al. suggested that the relationship between sleep-onset insomnia and frailty was mediated by a decline in physical performance[39]. In this vein, another study showed a notable connection of insomnia with frailty in the crude analysis (OR = 2.77, 95% CI = 1.36–5.67), although this association did not hold after adjusting for confounding factors (OR = 1.93, 95% CI = 0.81–4.61). Furthermore, subthreshold insomnia did not exhibit any association with frailty, neither in the unadjusted nor adjusted analysis [40].

Notably, good sleep was associated with a reduced risk of frailty (RR 1.42, 95% CI 1.31–1.54). [41]

With regard to the underlying mechanisms, the reviewed original studies did not provide any insights. However, some reviews suggested that disruptions in physiological processes, such as hormonal regulation, immune function, or inflammatory responses, may affect both sleep and frailty [6, 42]. Interestingly, two studies showed that sleep disturbances were associated with adverse muscle health and body composition, as well as premature aging [43, 44]. To summarize, there is sufficient evidence to suggest that sleep disturbances contribute to the development of frailty and that it is worth designing interventions to improve sleep with a view to preventing frailty.

### Frailty, comorbidity and urologic changes

Our search not only yielded results on nocturia, but also publications that dealt with the broader topic of LUTS (25 publications). The reviewed articles suggested that frailty is associated with worse LUTS and that frailty may promote LUTS and its severity through adverse lifestyle changes [11, 43, 45–47]. For example, in a study of 5979 men aged 65 years and above, moderate to severe LUTS were linked to increased odds of frailty. Men with moderate LUTS showed 1.55 times higher odds, and the odds of those with severe LUTS were 3.07 times higher compared to men without or with mild LUTS [48]. Here, too, the evidence was not sufficient to establish causality.

In the reviewed literature some interesting mechanistic observations were worth considering: Post-Void Residual (PVR), Void Percentage (%Void), and nocturnal urine volume (NUV) seem to be interesting parameters in the context of nocturia and frailty. Frail individuals seem to have higher PVR and NUV. Moreover, one publication found that nocturia is associated with low hip abductor strength [49], linking bladder function and muscle strength or sarcopenia, respectively. As to comorbidity, a high comorbidity burden and vascular risk factors predicted more severe or prevalent LUTS [43, 50], while exercise was shown to decrease LUTS [51].

### Consideration of frailty in the management of patients with BPH and LUTS

A handful of publications (4 articles) looked at the clinical management of patients with frailty, nocturia, and LUTS. It was consistently shown that there is poor LUTS improvement and more complications after TURP in frail older adults [52]. Therefore, several authors conclude that it is important to consider frailty right from the start in the management of patients with BPH and LUTS [48, 52–54].

## Discussion

The reviewed literature on nocturia and frailty emphasizes that it is an interesting and relevant topic, but there is a lack of high-quality evidence for a causal relationship. Only very few publications genuinely addressed the relationship between nocturia and frailty, and most of the evidence came from cross-sectional studies, with the known limitations. Our analysis indicates that interest in this topic has only recently increased. Most of the reviewed publications dated from the last 5 years – likely reflecting the recent overall increase in interest in frailty [55]. The fact that the publications came from very different disciplines emphasizes the complexity of the topic and the clinical and scientific fragmentation with regard to the treatment of the topic.

While the reviewed literature provides basal and indicative insights into the association between nocturia and frailty, further research is needed to underpin this relationship and to elucidate specific temporal trends and mechanisms that underlie the observed relationships. Understanding these connections could contribute to a better and integrated or even targeted approach to managing both, nocturia and frailty.

Based on the reviewed literature, we can say with certainty that nocturia and frailty are closely associated. They have shared risk factors, including sleep disturbances, multimorbidity, reduced physical activity, and physical decline [11, 24, 26, 34, 39, 56]. Furthermore, nocturia may be a risk factor for frailty – possibly through sleep disruption and functional decline. Nocturia inevitably causes sleep disruption, leading to fatigue, a decline in physical capabilities, and decreased cognitive function. This deterioration can set the stage for the onset of frailty. Likewise, disruption in the body's homeostasis and cleansing processes due to fragmented sleep cycles may be considered as an important cause of the emergence of nocturia-associated frailty [25, 53, 57–59]. Vice versa, functional decline and reduced physical activity, frailty, and sleep disturbances are risk factors for nocturia[51].

From a conceptual point of view, nocturia can be seen on the one hand as a bystander, i.e. having a more passive or indirect role (“indicator”) in the development of frailty [24, 25, 60]. On the other hand, there is some evidence for a more "active" role of nocturia on the path to frailty, in which it triggers or accelerates the development directly or via sleep disruption [60].

In fact, the disruption of the circadian rhythm may be a central mechanistic link between nocturia and frailty.[42, 61, 62]. The circadian rhythm is central for the homeostasis, and its disruption affects various signaling pathways, which ultimately may increase the odds of frailty [42, 61, 62]. Thrillingly enough, apart from the complex situation in older adults, there is a real situation in which the temporal sequence is clear: Shift workers face circadian disruption, as a result, they suffer from increased nocturia, and a higher risk of frailty. A recent study confirmed that people who had been exposed to shift work were at greater risk of premature frailty compared to those who only worked during the day. [63, 64]. Particularly women who worked in rotating shifts in their longest jobs were more likely to be classified as frail compared with those who worked only daytime [64]. Moreover, nocturia associated with circadian disruption has been linked to increased urine production and decreased storage function of the bladder [28, 43]. Furthermore, there is evidence that in older adults, age-related alterations in kidney function disrupt the circadian rhythm of diuresis (renal circadian biorhythms), contributing to nocturnal polyuria and LUTS [59]. Also, the identification of peripheral clock genes within the bladder and their involvement in contractile property of the bladder support that micturition is closely related to the circadian rhythm [63]. Supplementary Fig. 1 illustrates a possible version of a cycle of nocturia, circadian rhythm disruption and frailty. Whereas numerous studies have shown that disruption of the circadian rhythm (e.g. related to shift work) may increase risk for malignant, psychiatric, metabolic, cognitive and other diseases, the available evidence with respect to frailty later in life is scarce [65]. Further studies on nocturia and frailty should therefore examine the significance of circadian disruption [66].

Obviously, micturition and the regulation of body fluid compartments are closely connected. They are subject to complex neurohormonal complex regulations and are susceptible to disruption, e.g. as part of neurodegenerative processes (e.g. baroreflex or autonomic dysfunction) [67]. Indeed, the relation of extracellular water to total body water has recently received attention as an indicator of muscle quality, and has been proposed as a surrogate parameter for frailty [68]. This suggests another possible link between nocturia and frailty [69]. Indeed, frailty may be associated with a decline in the adaptability and modulation of the intricate neurohormonal systems regulating of micturition and homeostasis of body fluid compartments [68, 70, 71].

Like frailty, nocturia is strongly associated with age and is likely caused by a combination of diseases and failures in multiple systems, resulting in a loss of corresponding functions and the ability to compensate. Therefore, nocturia may be considered a geriatric syndrome, too [11, 72].

### Therapeutic approaches

Known strategies to reduce nocturia include avoiding large amounts of fluids, especially caffeine and alcohol, in the hours before bedtime, voiding before bedtime, elevating the legs during the day and/or before bedtime or using compression stockings, medication adjustment, physical activity, exercise, hygiene, etc. Another promising avenue for intervention lies in circadian-based strategies, exemplified by the innovative concepts of time-restricted eating, targeted light exposure or scheduled exercise [73]. Such interventions offer actionable approaches to sustain the circadian regulation of physiology, metabolism, and behavior [73]. In managing frail and pre-frail older adults, circadian optimization may offer a simple and holistic approach to maintain and improve health and well-being, as it not only addresses specific conditions such as nocturia, but also has broader implications for healthy ageing [74, 75].

### Strengths and limitations

The study has both strengths and limitations. A reproducible and systematic approach was taken, from the literature search to screening and data extraction, which was conducted by two independent reviewers.

Regarding limitations, we acknowledge that our search was restricted to selected, albeit the most important databases, which may have limited the number of studies we were able to identify. Additionally, we only considered publications in German and English.

## Conclusion

In conclusion, the findings emphasize the increasing interest and interdisciplinary nature of research into the relationship between frailty and nocturia. This is an interesting and relevant topic, but there is a lack of good quality evidence of a causal relationship. Further research is required to enhance understanding, establish causality, and identify potential therapeutic approaches. The complex interplay between nocturia, lower urinary tract symptoms, sleep, frailty, and functional decline highlights the intricate links within geriatric syndromes. Recognizing the multifactorial nature of these conditions makes holistic interventions crucial to break the vicious circle and counteract frailty.

### Supplementary Information

Supplementary Material 1.

## Data Availability

All data generated or analysed during this study are included in this published article and its supplementary information files.
