# Supplementary material for: Nocturia and frailty in older adults: a scoping review
Source: BMC Geriatr. 2024 Jun 6;24:498. doi: 10.1186/s12877-024-05049-3 (PMC11155172; doi:10.1186/s12877-024-05049-3)
Supplement: Supplementary file 1 — Supplementary Material 1. [file 12877_2024_5049_MOESM1_ESM.pdf]

Nocturia and frailty in older adults: a scoping review

Yulia Komleva[1,2], Maik Gollasch[1,2], Maximilian König[1,2*]

[1]Klinik und Poliklinik für Innere Medizin D – Geriatrie, Universitätsmedizin Greifswald, Greifswald, Germany

[2]Altersmedizinisches Zentrum, Kreiskrankenhaus Wolgast, Wolgast, Germany

*corresponding author

Supplementary material

Supplementary Figure 1.

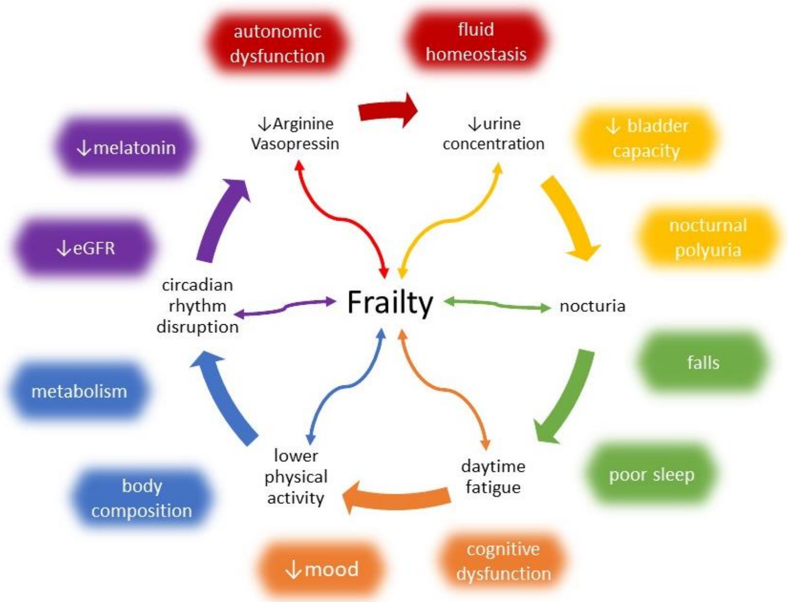

Supplementary Figure 1. Cycle of Nocturia, Circadian Rhythm Disruption, and Frailty

Illustration of a potential cycle involving nocturia, circadian rhythm disruption, and frailty. The diagram depicts a closed loop of interrelated influencing factors and consequences. Starting at the top, circadian rhythm disruption impairs Arginine Vasopressin signalling, which causes nocturnal polyuria and nocturia due to decreased urine concentration, contributing to daytime fatigue and reduced physical activity, exacerbating

circadian rhythm disruption. There are numerous, diverse influencing factors and consequences, encompassing falls, poor sleep quality, mood disorders and cognitive impairments, alterations in body composition, metabolic changes, reduced estimated glomerular filtration rate (eGFR), diminished melatonin production, autonomic dysfunction, and disturbances in fluid homeostasis.

Supplementary Table 1. Structured overview of all included studies

	**Author (Year)**	**DOI**	**Study Design, number of participants, age**	**Frailty**	**Nocturia**	**LUTS**	**Sleep**	**Main results messages**	**Comments**
1	Nguyen et al. (2021)	10.1111/ajag.13002	Cross-sectional, n=151, mean age 83 years	No	Yes	Yes	No	Nocturia increases with age, and is associated with daytime incontinence, no difference between men and women	
2	Konishi et al. (2021)	10.1111/luts.12326	Cross-sectional, n=723, mean age 64 years	Yes	Yes	Yes	Yes	Participants with nocturia ≥ times per night had significantly higher PSQI (median 3 vs 4, $p < 0.001$) than those with OABSS < 6.	
3	Soma et al. (2018)		Cross-sectional, n= 843 (338 male, 505 female)	Yes	Yes	Yes	No	Frailty is potentially associated with LUTS	No full text

4	Bauer et al. (2020)	10.1111/jgs.16766	Cross-sectional, n= 5979, only men, age 65+	Yes	Yes	Yes	No	LUTS increased odds of frailty ("co-occur")	
5	Decalf et al. (2019)	10.1002/nau.24118	Congress abstract, mean age 79 years	Yes	Yes	Yes	No	Frail adults have higher PVR and more nocturia	No full text
6	Gibson and Wagg (2014)	10.2217/cpr.14.38	Review	No	Yes	Yes	No	Vascular risk factors increase LUTS<br><br>Exercise reduces nocturia	
7	Gordzheladze et al. (2023)	10.1007/s40520-023-02442-7	Conference abstract, n = 46, mean age 73.02 ± 8.37 years	Yes	Yes	No	No	The presence of geriatric syndromes is associated with an increased risk of falls and the occurrence of osteoporotic fractures in elderly patients. Nocturia was detected in 26 (56.52%) patients, stress urinary incontinence in 4 (8.70%), combined urinary disorders in 3 (6.52%)	No full text
8	Haddad et al. (2022)	10.1016/j.gerinurse.2022.09.009	Cross-sectional, n=80, mean age 89 years	No	Yes	No	Yes	Nocturia is associated with increased risk of poor sleep quality	
9	Dutoglu E et al. (2019)	10.1016/j.archger.2019.103917	Cross-sectional study, n=858, only women,	Yes	Yes	No	Yes	The presence of polypharmacy, frailty was significantly associated with ≥ 4 voids/night	

			mean age 74.1 ± 8.0 years						
10	Decalf et al. (2021)	10.1007/s41999-021-00585-2	Cross-sectional, n= 80, 77% women, mean age 88 ± 7 years	Yes	Yes	No	No	No statistically significant associations were found between cognitive frailty risk and the presence of nocturia	
11	Soma et al. (2020)	10.1111/luts.12292	Cross-sectional, n= 710, mean age 67,5 ± 5.8 years	Yes	Yes	Yes	No	IPSS ≥8 was significantly associated with FDS (P < .001; OR 2.13), but not FFP and mFI.<br>The OABSS ≥6 was significantly associated with FDS alone (P = .002; OR 2.07).<br>Not all frailty assessment tools showed a positive association with the severity of LUTS	
12	Monaghan et al. (2020)	10.1007/s40520-019-01416-y	Cross-sectional, n= 158, only men, ≥65 years	Yes	Yes	No	No	NUV did not differ by frailty (FI) (p=0.333)	
13	Hatakeyama et al. (2019)	10.1016/S1569-9056(19)31132-7	Congress abstract, cross-sectional, 896 men and 1325 women	Yes	Yes	No	No	Nocturia was significantly associated with falls, whereas mFI did not show significant association. Nocturia might have a	No full text

								greater impact on falls than frailty.	
14	Fitzgerald et al. (2009)	10.1002/nau.20808	Cross-sectional, n =1496, only men, aged 60 and older	No	Yes	No	No	Nocturia (3+ per night) is highly prevalent in older Puerto Rican men and predicts significantly increased 2-year mortality	
15	Bower WF et al. (2017)	10.1002/nau.23000	Systematic Review	No	Yes	Yes	Yes	Insomnia was independently predicted by nocturia	
16	Hunter et al. (2012)	10.1002/nau.22287	Longitudinal, n = 104, only women, mean age 84.3 years, Congress abstract	No	No	Yes	No	Correlations between total ICIQ-LUTS scores and the QoL score were modest (r = 0.54, 0.57 and 0.65 at baseline, 3 and six months respectively) but significant (p = <0.000) at all data points	
17	Irie et al. (2023)	10.1111/ggi.14633	Cross-sectional, n = 405, mean age 73.1±6.25 years	Yes	No	Yes	No	Factors related to frailty according to multivariate analysis were female sex (p=0.001), older age (p< 0.001), and LUTS (p< 0.033)	
18	(Bauer et al. (2023)	10.1111/jgs.18171	Longitudinal,	Yes	No	Yes	No	More severe phenotypic frailty was associated with non-linear increases in LUTS severity in older men over	

			n = 3235, only men, age ≥71 years					time, independent of age and comorbidities. Results suggest LUTS and frailty share an underlying mechanism that is not targeted by existing LUTS interventions.	
19	Ren et al. (2023)	10.3389/fmed.2023.1185539	Longitudinal,<br>n =567, mean age 75.2±8.9 frail	Yes	No	Yes	No	Frailty is a strong predictor of poor outcomes after TURP<br>Frail individuals are at higher risk of adverse postoperative LUTS and HRQOL outcomes	
20	Nishii (2021)	10.5213/inj.2142042.021	Review	Yes	No	Yes	No	Frailty could contribute to LUTS by changing physical activities, prolonged sitting time and low physical activity level with the development of LUTS among middle-aged men.	
21	Karim and Rantell (2021)	10.7748/nop.2021.e1305	Review	No	No	Yes	No	LUTS such as urinary incontinence have a significant negative effect on people's quality of life	no full text
22	Bower et al. (2021)	10.1002/nau.24746	Cross-sectional, n = 64, mean age 82.6± 7.7	Yes	No	Yes	No	Physical function parameters, skeletal muscle mass, Cumulative Illness	

			years, 59.9% female					Rating, Charlson Comorbidity Index, Frailty score and self-reported constipation did not differ by presence or absence of clinically relevant nocturia.	
23	Bauer et al. (2021a)	10.1016/j.urology.2020.09.041	Cross-sectional, n =2206, only men, mean age 79 ± 7 years	Yes	No	Yes	No	Compared to non-LUTS conditions, OAB (odds ratio (OR)=2.62, 95% CI 1.74, 3.93), BPH (OR=1.70, 95% 1.14, 2.55), and mixed OAB/BPH (OR=1.82, 95% 1.14, 2.92) were all associated with increased odds of slow TUG time, a surrogate of frailty. Frailty is common among older men with LUTS and should be considered during the initial urological evaluation.	
24	Zuo et al. (2021)	10.1002/nau.24638	Cross-sectional study, n=200, only women, median age 60 (IQR 49-69)	Yes	No	Yes	No	Higher EFS scores were associated with urinary leakage (p=0.047), and difficulty with bladder emptying (p=0.053).	

25	Zuo et al. (2020)	10.1002/nau.24307	Cross-sectional, n =179, only women, mean age 67.5 years	Yes	No	Yes	No	Women with higher EFS were more likely to complain of worse LUTS (OAB-V8, 95% CI [0.01,0.08]; UDI- 6, 95% CI [0.47, 5.47]). Median TUG time was 9.24 seconds (IQR 8-12; n=138). TUG was highly correlated to EFS score (P< 0.001). Frailty is associated with worse LUTS and should be considered in the diagnosis and management of urinary symptoms.	
26	Matsuo et al. (2019)	10.1097/01.JU.0000557200.85476.8d	Congress abstract, cross-sectional study, n=182	No	No	Yes	No	VFA was positively correlated with severity of slow urinary stream.	No full text
27	Gibson et al. (2018)	10.1002/nau.23295	Review	No	No	Yes	No	Evidence of association between falls and LUTS in older adults, with estimates of odd ratios for falls in the presence of LUTS ranging between 1.5 and 2.3. Falls and LUTS are both symptoms of frailty and have many common causes. Gait, balance, and continence are all processes requiring cognitive input, and the	

								concept of dual tasking may be a further link.	
28	Ozaki et al. (2023)	10.1111/iju.15187	Longitudinal, n=247, mean age 60 years	Yes	No	Yes	No	5i-mFI score was significantly associated with the severity of LUTS in 2014 to 5i-mFI in 2019 but not with 5i-mFI in 2014 to the severity of LUTS in 2019. The effect of LUTS on frailty might be greater than the effect of frailty on LUTS.	
29	Bauer et al. (2023)	10.1111/jgs.18171	Longitudinal, n=3025, only men, age ≥71 years	Yes	No	Yes	No	LUTS severity was not associated with the risk of new life-space mobility restrictions.	
30	Bauer et al. (2022)	10.1111/jgs.17633	Longitudinal, n=2716 men, age ≥71 years	Yes	No	Yes	No	LUTS severity is associated with incident mobility and ADL limitations among older men.	
31	Bellos et al. (2022)	10.4081/aiua.2022.3.345	Review	Yes	No	Yes	No	BPH has a strong association with frailty and increasing age.	No full text
32	Bauer et al. (2021c)	10.1001/jamanetworkopen.2021.34427	Randomized controlled trial, n=3047, only men, mean	Yes	No	Yes	No	Frailty is independently associated with greater risk of both clinical BPH progression and serious adverse events.	

			age, 62.6±7.3 years						
33	Bauer et al. (2021b)	10.1111/jgs.17115	Longitudinal, n=3235, only men, age >65 years	Yes	No	Yes	No	Physical frailty and LUTS severity increased concurrently among older men without clinically meaningful LUTS at baseline.	
34	Gibson (2021)	10.1016/j.ogrm.2021.01.003	Review	No	No	Yes	No	Comorbidity causes LUTS and decreased QoL.	
35	Yoshida et al. (2020)	10.1016/S2666-1683(20)33119-0	Cross-sectional, n= 390, mean age 75.5±5.30 years	Yes	No	Yes	No	There is a sign association (OR 2,84) between LUTS and frailty.	
36	Painter and Suskind (2019)	10.1007/s11884-019-00562-3	Review	Yes	No	Yes	No	Frailty is important to consider in therapy.	
37	Monaghan et al. (2019)	10.1007/s40520-019-01416-y	Cross-sectional, n=158, only men, age ≥ 65 years	Yes	Yes	No	No	Frailty is associated with increased nocturnal urine production.	
38	Suskind (2017)	10.1007/s11934-017-0720-9	Review	Yes	No	Yes	No	Frailty and LUTS both increase with age, frailty is important to consider when caring for older adults with LUTS.	

39	Coll-Planas et al. (2008)	10.1007/s00391-008-0563-6	Review	Yes	No	Yes	No	Disablement-process-model:<br>UI -> functional impairment	
40	Athavale et al. (2019)	10.1111/jgs.15898	Congress abstract, cross-sectional, n =514, age was categorized as <65 and ≥65 years of age	Yes	No	Yes	No	frailty was associated with several elements of OAB burden.	No full text
41	Nuotio et al. (2019)	10.1007/s40520-018-0946-5	Cross-sectional, n=409, only women, age ≥65 years	No	No	Yes	No	PVR deserves to be included in the CGA of frail older patients, including women.	
42	Gibson and Wagg (2017)	10.1038/nrurol.2017.53	Review	No	No	Yes	No	The prevalence of urinary incontinence and other LUTS increases in association with increasing age.	
43	Nuotio and Luukkaala (2016)	ISSN 1878-7649	Cross-sectional, n=409, only women, age ≥65 years	No	No	Yes	No	PVR measurement may be included in the CGA especially in frail older patients.	
44	Gibson and Wagg (2014)	10.2217/cpr.14.38	Review	Yes	No	Yes	No	Urinary incontinence and lower urinary tract symptoms are highly prevalent in older adults,	

								and are strongly associated with frailty.	
45	Yoshikoshi S et al. (2023)	10.1159/000533418	Cross-sectional, n=575, mean age 65.6 years	Yes	No	No	Yes	Poor sleep is associated with increased odds of frailty (OR 1,12).	
46	Frohnhofen H et al. (2023)	10.1007/s00391-023-02219-7	Review	Yes	No	No	Yes	The risk of frailty is increased in individuals with sleep disturbances.	
47	Arias-Fernández L et al. (2021)	10.1093/fampra/cmaa085	Cross-sectional, n=392, age ≥65 years	Yes	No	No	Yes	Poor self-reported sleep quality, but not sleep duration, was associated with an increased frequency of physical frailty (OR 2.90).	
48	Sun XH et al. (2020)	10.1186/s12877-019-1407-5	Cross-sectional, n=1726, mean age 77.6 ± 3.9 years	Yes	No	No	Yes	Poor sleep quality (PSQI> 5) was associated with higher odds of frailty (OR = 1.78) and pre-frailty (OR = 1.51).	
49	Kumar et al. (2019)	10.3390/pharmacy7040143	Cross-sectional study, n=151, mean age 74.47 ±8.30 years	Yes	No	No	Yes	An increased anticholinergic cognitive burden was associated with frailty (p = 0.031), sleep latency (p = 0.007), and sleep disturbances (p = 0.015).	Care Home Residents

50	Kang et al. (2019)	10.1007/s12603-018-1109-2	Cross-Sectional, n=1168, 70 to 84 years old	Yes	No	No	Yes	Prolonged sleep latency (≥60 minutes) in men and long sleep duration (>8hr per night) in women were each independently associated with higher odds of frailty.	
51	Ensrud KE et al. (2009)	10.1111/j.1532-5415.2009.02490.x	Cross-Sectional, n=3133, mean age 76.4 ± 5.6 years	Yes	No	No	Yes	Multivariable odds ratio (MOR) 1.28, 95%CI 1.09–1.50), sleep efficiency <70% (MOR 1.37, 95% CI 1.12–1.67), sleep latency ≥60 minutes (MOR) 1.42, 95% CI 1.10–1.82), and sleep disordered breathing (respiratory disturbance index ≥15, MOR 1.38, 95% CI 1.15–1.65) were each independently associated with an increased odds of worse frailty status.	
52	Balomenos et al. (2021)	10.1016/j.jamda.2020.08.012	Cross-sectional, n= 1984, age ≥65 years	Yes	No	No	Yes	Sleep quality is correlated with frailty.	No access to full text

53	Alshehri et al. (2019)	10.1093/sleep/zsz067.706	Cross-sectional, n=108, age 65-85 years	Yes	No	No	Yes	Sleep disturbances are associated with frailty.	
54	Piovezan et al. (2015)	10.1016/j.arr.2015.07.003	Review	Yes	No	No	Yes	Mechanistic links of bad sleep and muscle health/body composition.	
55	Ensrud et al. (2012)	10.1016/j.sleep.2012.04.010	Longitudinal, n=2505, only men, mean age 67 years	Yes	No	No	Yes	PSQI/bad sleep quality are associated with frailty, and wakefulness.	
56	Wen et al. (2023)	10.1002/brb3.2793	Systematic review and meta-analysis	Yes	No	No	Yes	Sleep disorders are independently associated with frailty (AIS>PSQI).	
57	Madan Jha (2023)	10.1016/j.nbas.2022.100057	Opinion	Yes	No	No	Yes	Circadian system instability<br>Role of melatonin<br>Growth hormone<br>Sleep, TSH, CVD, disability, cognitive function, frailty, low daytime light exposure, hormone,<br>Risk factors for frailty<br>Inflammation	

58	Çakmak (2022)	10.1007/s41999-022-00711-8	Congress abstract, cross-sectional, n= 762	Yes	No	No	Yes	Poor sleep is associated with frailty.	
59	Shen et al. (2022)	10.1186/s12877-022-03572-9	Congress abstract, cross sectional, n = 540	Yes	No	No	Yes	Poor sleep + pain are associated with frailty (CFS).	No full text
60	Gomez et al. (2022)	10.1007/s40520-022-02125-9	Cross-sectional, n = 17425, 98% men, mean age 75.53± 8.03 years	Yes	No	No	Yes	Use of benzodiazepines is associated with frailty.	
61	Griffiths J et al. (2023)	10.3390/nu15132849	Cross-sectional, n = 408, mean age 70 ± 5,48 years	Yes	No	No	Yes	Relationship between frailty and sleep in the context of COVID-19.	
62	Chang YH et al. (2023)	10.1016/j.sleep.2023.06.016	Cross-sectional, n=1268, age ≥ 60 years	No, intrinsic capacity	No	No	Yes	Sleep and intrinsic capacity are associated.	No full text
63	Wu CS et al. (2023)	10.1016/j.jagp.2023.01.028	Longitudinal, n = 4744, mean	Yes	No	No	Yes	Insomnia and sedative-hypnotic use were independently associated	No full text

			age 69.4 ± 8.2 years					with increased frailty. The implementation of nonpharmacological treatments to attenuate insomnia may reduce frailty rates.	
64	Carvalhas-Almeida C et al. (2023)	10.1007/s40520-022-02310-w	Review	Yes	No	No	Yes	Crucial role of biol. clock in the maintenance of whole-body homeostasis → disruption promotes biol. aging and frailty.	
65	Zhu Y et al. (2022)	10.1186/s12916-022-02557-0	Longitudinal study, n=23847, 30-79 years	Yes	No	No	Yes	Good sleep is associated with reduced HR of frailty transition (HR 1,41).	
66	Liu S et al. (2022)	10.3389/fpubh.2022.963105	Cross-sectional, n=1,206, mean age 77.32 ± 8.87 years	Yes	No	No	Yes	Poor sleep quality (OR 2.53; 95% CI 1.78-3.59; P < 0.001) and long nap duration (OR 1.77; 95% CI 1.19-2.64; P = 0.003) were associated with higher odds of cognitive frailty, but short nap duration (OR 0.60; 95% CI 0.40-0.89; P = 0.012) was associated with lower prevalence of cognitive frailty.	

67	Xu X et al. (2022)	10.1186/s12877-022-03285-z	Cross-sectional, n=247, 60–74 years old	Yes	No	No	Yes	Logistic regression analysis of factors related to frailty: Poor sleep quality PSQI > 7 OR(95% CI)2.68 (1.04, 6.91) P=0.041<br><br>Logistic regression analysis of factors related to sleep quality: frailty OR(95% CI) 2.14 (1.15,3.99), P= 0.017.	
68	Nemoto Y et al. (2021)	10.1016/j.archger.2021.104519	Longitudinal, n=3844, mean age 74.0± 6.4 years	Yes	No	No	Yes	The direction of association between insomnia and frailty may vary by sex.<br><br>Poor sleep predicted the onset and worsening of frailty during follow up in men (standardized coefficient [95% confidence interval]: 0.076 [0.045, 0.107])<br><br>Frailty predicted severe insomnia symptoms in women (0.074 [0.044, 0.104])	
69	Moreno-Tamayo K et al. (2021)	10.2147/NSS.S320192	Cross-sectional, n=1643, mean age 67.1 ± 5.9 years	Yes	No	No	Yes	In community-dwelling older adults, both the risk of OSA and insomnia conferred	

								greater odds of presenting frailty in women.<br><br>The association between risk of OSA and frailty was observed in women (odds ratio (OR) = 1.84, 95% confidence interval (CI) 1.05, 3.22), but not in men (OR = 1.19, 95% CI: 0.65, 2.19). Insomnia was significantly associated with frailty in women (OR = 2.38, 95% CI: 1.35, 4.20) and in men (OR = 1.88, 95% CI: 1.01, 3.52). Neither sleepiness nor sleep duration was associated with frailty.	
70	Fan J et al. (2022)	10.1111/jocn.15893	Cross-sectional, n= 454, mean age 68.0±6.1	Yes	No	No	Yes	Insomnia was associated with frailty, after adjusting for sociodemographic characteristics and comorbidity OR of 6.863 (95% CI: 4.237-11.116).	.
71	Tang JY et al. (2021)	10.1016/j.sleep.2021.05.039	Cross-sectional n=345, mean age (frail group): 79.8±0.51	Yes	No	No	Yes	The prevalence of sleep-onset insomnia was higher in frail participants than their prefrail counterparts (48% vs. 34%, aOR = 1.73; 95% CI 1.09, 2.76; p = 0.02), but	

			(prefrail group): 82.8±0.61					there was no significant difference in the prevalence of sleep-maintenance insomnia (61% vs. 54%). The association between sleep-onset insomnia and frailty was explained by reduced physical performance, but not depression and level of physical activity. Sleep-maintenance insomnia was not associated with frailty.	
72	Hui Y et al. (2021)	10.1097/MEG.0000000000000002231	Cross-sectional, n=105, mean age 61.6 ± 9.5 years	Yes	No	No	Yes	PSQI score was markedly associated with the Frailty Index (β = 0.012; 95% CI, 0.006-0.018; P < 0.001), and remained significantly associated with frailty phenotype in multivariate adjustment (β = 0.010; 95% CI, 0.004-0.015; P = 0.001)	cirrhotic patients
73	Liu M et al. (2021)	10.1016/j.ijnurstu.2021.103873	Cross-sectional, n = 7,609, ≥65 years	Yes	No	No	Yes	Independent associations with pre-frailty and frailty were found for pain (odds ratio [OR]: 1.81, 95% CI: 1.60, 2.04), difficulty initiating sleep (OR: 1.23, 95% CI: 1.04, 1.46) and	

								depressive symptom (OR: 2.29, 95% CI: 1.85, 2.84).	
74	Ma L et al. (2021)	10.1136/bmjopen-2020-043062	Cross-sectional, n= 5823, age 60–98 years	No,<br>Intrinsic capacity	No	No	Yes	Insomnia and urinary incontinence, were related to IC decline. IC decline was independently associated with risk of frailty, disability, falls, fractures and immobility.	
75	Pachołek A et al. (2020)	PMID: 35023893	Cross-sectional, n=438, mean age 75.6 ± 7.9 years	Yes	No	No	Yes	There is an association between Insomnia and Frailty scales [OR =2.93 (1.94-4.42); p <0.001].	
76	Çavuşoğlu Ç et al. (2021)	10.3906/sag-2001-168	Cross-sectional study, n=100, mean age 84 years	Yes	No	No	Yes	The PSQI score (odds ratio [OR] of 1.308, 95% confidence interval [CI]: 1.092–1.566, P = 0.004), was found to be independently associated with frailty.	
77	Xie B et al. (2021)	10.1111/hsc.13092	Cross-sectional, n=1585, mean age 81.38 ± 4.72 years	Yes	No	No	Yes	Sleep problems (insomnia occasionally, OR = 1.84, 95% CI = 1.07-3.17; insomnia every day, OR = 2.38, 95% CI = 1.33-4.26) significantly associated with the co-occurrence of physical frailty and cognitive impairment.	

78	Moreno-Tamayo K et al. (2020)	10.1016/j.maturitas.2020.03.005	Cross-sectional study, n=493, mean age 70.1 ± 5.6 years	Yes	No	No	Yes	For women, reporting low sleep quality was highly associated with frailty in the model adjusted for covariates (OR = 3.34; CI 95 % 1.37–8.15), but not in men (OR = 2.71; CI 95 % 0.64–11.50).<br><br>Short sleep duration, specifically sleeping < 5 h, was associated with the presence of frailty (OR = 4.03; CI 95 % 1.83–8.88),	
79	Tang JY et al. (2021)	10.1016/j.sleep.2021.05.039	Systematic review	Yes	No	No	Yes	Six cross-sectional studies and one longitudinal study: this review has identified consistent evidence on the relationship between perceived sleep quality and frailty.	
80	Morgan K and Hartescu I (2019)	10.1016/j.sleep.2018.11.008	Longitudinal, n= 960, age ≥74 years	Yes	No	No	Yes	The lowest physical activity quintile and very slow walking speed significantly increased mortality risk (HR: 1.79; 95% CI: 1.40–2.30; HR: 1.41; 95% CI: 1.15–1.73 respectively)	

								When indices of frailty were added to the model, hazard ratios for long sleep duration and hypnotic drug became non-significant.	
81	Lee DR et al. (2018)	10.1186/s12875-018-0851-1	Cross-sectional, n=4551, mean age 73.3 years	Yes	No	No	Yes	Frail older adults were more likely than non-frail to have insomnia (36.4% vs. 8.8%).	
82	Moreno-Tamayo K et al. (2017)	10.1111/ggi.13111	Cross-sectional, n= 591, 76.3 ± 3.3 years	Yes	No	No	Yes	Sleep complaints were associated with increased odds of frailty in women (OR 3.24, 95% CI 1.34-7.84), but not in men (OR 0.76, 95% CI 0.23-2.51).	
83	Leigh L et al. (2016)	10.1177/0898264315624907	Longitudinal, only women, age 70 to 90 years	No	No	No	Yes	Patterns of sleeping difficulty were associated with arthritis, heart disease, respiratory conditions, diabetes, osteoporosis, and hypertension.	
84	Vaz Fragoso CA et al. (2009)	10.1111/j.1532-5415.2009.02522.x	Cross-sectional, n=374, mean age 84.3 years	Yes	No	No	Yes	Clinical insomnia was significantly associated with frailty in the unadjusted analysis (OR=2.77, 95% CI=1.36-5.67) but not the adjusted analysis (OR=1.93, 95% CI=0.81-4.61)), and subthreshold insomnia was	

								not associated with frailty in the unadjusted or adjusted analysis.	
85	Cochen V et al. (2009)	10.1007/s12603-009-0030-0	Review	No	No	No	Yes	Nocturnal sleep disruption and daytime sleepiness are the results of sleep/wake circadian rhythm disorders, environmental, psychological, and iatrogenic factors.	
86	LeMaster et al. (2023)	10.1177/10815589221142328	Experimental study in mice	No	No	No	Circadian control in mice	Scheduled exercise could increase expression of AVP, and relieve symptoms of nocturia.	
87	Bliwise et al. (2019)	10.1016/j.urology.2019.07.005	Review	No	Yes	No	Yes	Nocturia is associated with disruption of sleep → daytime fatigue, cognitive impairment, mood alterations, increased susceptibility to disease, decreased work performance, dizziness, an increased risk of falls, depression, and mortality.<br>The lower estrogen levels, the more nocturia.	

Abbreviations: LUTS, lower urinary tract symptoms; OABSS, Overactive Bladder Symptom Score; (a)OR, (adjusted) odds ratio; CI, confidence interval; HR, hazard ratio; IC, intrinsic capacity; PSQI, Pittsburgh Sleep Quality Index; OSA, obstructive sleep apnea; PVR, post-void residual volume; IPSS, International Prostate Symptom Score; FDS, frailty discriminant score; mFI, modified frailty index; NUV, nocturnal urine volume; FI, frailty index; ICIQ, International Consultation on Incontinence Questionnaire; TURP, Trans urethral resection of the prostate; HRQOL, health related Quality of Life; OAB, overactive bladder; BPH, benign prostate hyperplasia; EFS, Edmonton Frail Scale; TUG, Timed-Up and Go; VFA, visceral fat accumulation; ADL, activities of daily living; UI, urinary incontinence; CGA, comprehensive geriatric assessment; AIS, Athens insomnia scale; ISI, Insomnia severity index; TSH, thyroid-stimulating hormone; CVD, cardiovascular disease; CFS, clinical frailty scale; AVP, Arginine vasopressin.
